# Adoption of clinical pharmacist roles in primary care: longitudinal evidence from English general practice

**DOI:** 10.3399/BJGP.2024.0320

**Published:** 2025-01-28

**Authors:** Michael Anderson, Igor Francetic

**Affiliations:** Health Organisation, Policy, Economics (HOPE), Centre for Primary Care & Health Services Research, The University of Manchester, Manchester, UK; LSE Health, Department of Health Policy, London School of Economics and Political Science, London, UK.; HOPE, Centre for Primary Care & Health Services Research, The University of Manchester, Manchester, UK; Department of Business Economics, Health and Social Care, University of Applied Sciences and Arts of Southern Switzerland, Manno, Switzerland.

**Keywords:** clinical pharmacy, drug prescription, healthcare quality, pharmacist, prescribing, primary care

## Abstract

**Background:**

Over the past decade, the number of clinical pharmacists working within multidisciplinary teams in English general practices has expanded.

**Aim:**

To examine changes in quality of prescribing after the adoption of clinical pharmacist roles in English general practices.

**Design and setting:**

Longitudinal cohort study in English general practice.

**Method:**

Two-way fixed-effects regression was used to compare differences in prescribing indicators in general practices with and without pharmacists between September 2015 and December 2019.

**Results:**

Between September 2015 and December 2019, the proportion of practices employing a clinical pharmacist increased from 236/7623 (3.1%) to 1402/6836 (20.5%). Clinical pharmacist implementation resulted in statistically significant reductions in total costs of medicines per 1000 patients (−0.85%, 95% confidence interval [CI] = −1.50% to −0.21%), the total number of opioid prescriptions per 1000 patients (−1.06%, 95% CI = −1.82% to −0.29%), and the average daily quantity of anxiolytics per 1000 patients (−1.26%, 95% CI = −2.40% to −0.12%). Clinical pharmacist implementation also resulted in reductions in the total number of prescriptions per 1000 patients (−0.58%, 95% CI = −1.30% to 0.13%) and the total number of antibiotic prescriptions per 1000 patients (−0.51%, 95% CI = −1.30% to 0.27%) that trended towards statistical significance. There were no statistically significant differences in the share of broad-spectrum versus narrow-spectrum antibiotics (0.02%, 95% CI = −0.07% to 0.11%) and the oral morphine equivalence of high-dose opioids (>120 mg per 24 h) per 1000 patients (1.19%, 95% CI = −0.46% to 2.85%).

**Conclusion:**

This analysis is limited by practice-level data but supports the hypothesis that clinical pharmacist implementation results in improvements in prescribing quality.

## Introduction

Over the past decade there have been several policy initiatives that have expanded the clinical pharmacist workforce in English general practice. The *General Practice Forward View*, published in 2016, committed to employing 1500 clinical pharmacists to work in general practice over 5 years.^1^ The subsequent Additional Roles Reimbursement Scheme, launched in 2019, further expanded the clinical pharmacy workforce with the aim of recruiting six clinical pharmacists for each primary care network (PCN) by 2024.^2^^,^^3^ As of September 2023, there are over 6500 clinical pharmacists working in general practice in England either employed by a PCN or directly by a general practice.^4^

The ambition by NHS England has been to expand the number of pharmacists working in general practice with responsibility for optimising medicines management, conducting medication reviews, and independent prescribing and deprescribing.^5^^,^^6^ When the authors refer to clinical pharmacists in this article, they are referring specifically to pharmacists working within multidisciplinary teams in general practice and consulting directly with patients.^7^ This differs from the role of community pharmacists within pharmacies as dispensers and retailers of medicines, alongside providing other NHS services.^8^

International evidence on the impact of pharmacists working within primary care teams on prescribing outcomes is mixed, with some evidence of significant reductions in the number of prescriptions and medication costs per patient.^9^^,^^10^ Focusing on England, a pilot evaluation of clinical pharmacists working within general practices found evidence that clinical pharmacists could improve access to appointments for people with long-term conditions, facilitate deprescribing, and reduce medication errors.^7^^,^^11^ Further analysis of practice-level data has found significant associations between the number of allied health professionals (including clinical pharmacists) working in general practice and fewer prescriptions of broad-spectrum antibiotics and costs per item prescribed.^12^ However, the analysis did not examine the impact of the number of clinical pharmacists independently. Additional analysis of a broader set of prescribing indicators would contribute to a more comprehensive understanding of the influence of clinical pharmacist implementation in English general practice on quality of prescribing. This study addresses this gap in the literature by examining changes in quality of prescribing following adoption of clinical pharmacist roles in English general practice.

**Table:** How this fits in

There has been rapid expansion of clinical pharmacists working within multidisciplinary teams in general practice in England over the past decade that has not been subject to robust evaluation. This study examined the impact of the first wave of clinical pharmacist expansion in general practice, between September 2015 and December 2019, when clinical pharmacists were directly employed by practices. The study found that clinical pharmacist implementation was associated with reduced prescribing costs per patient and reductions in the total number of items, opioids, antibiotics, and anxiolytics prescribed per patient. Future research is needed to evaluate the second wave of clinical pharmacist implementation in general practice, when they are employed by primary care networks.

## Method

### Study cohort

The analysis in this study focused on all general practices in England between September 2015 and December 2019. During this period, clinical pharmacists were directly employed by practices and therefore it is possible to attribute their presence within specific practices. Mixed employment of clinical pharmacists, either directly by general practices or by PCNs, begins beyond this period. This time period was also chosen as it avoids any influence on prescribing created by the COVID-19 pandemic.

Information on the primary care workforce involved in direct care was obtained from NHS England,^4^ and practice-level information on population characteristics such as age, gender, and deprivation from the Office for Health Improvement and Disparities.^13^ The study focused exclusively on the number of clinical pharmacists working in general practice in the primary analysis, and did not include pharmacy technicians or advanced pharmacy practitioners as these roles have different responsibilities to clinical pharmacists.^5^ However, these roles were included in a supplementary analysis to ascertain if this changed the results.

Pharmacy technicians undertake some tasks understood as not requiring professional or clinical judgement such as patient counselling regarding safe use of medicines, medicines reconciliation, and taking drug histories under the supervision of a clinical pharmacist.^14^ Advanced pharmacy practitioners are autonomous clinicians that are typically independent prescribers and typically see patients with minor ailments and conduct complex medicines reviews.^15^

### Study outcomes

In total, data were extracted on seven different prescribing indicators. Data were retrieved on a quarterly basis from the NHS Business Service Authority (BSA) English Prescribing Dataset for the following indicators:

- total number of prescriptions per 1000 patients;
- total costs of medicines per 1000 patients;
- total number of antibiotic prescriptions per 1000 patients;
- share of broad-spectrum versus narrow-spectrum antibiotics;
- total number of opioid prescriptions per 1000 patients;
- oral morphine equivalence of high-dose opioids (>120 mg per 24 h) per 1000 patients; and
- average daily quantities (ADQs) of anxiolytics per 1000 patients.

Relevant British National Formulary (BNF) codes were identified for these indicators from the Open Prescribing website (https://openprescribing.net). The relevant BNF codes used to extract each indicator are also contained in Supplementary Table S1. ADQ is a unit of measure that refers to actual prescribed daily doses for a medicine, which differs from defined daily doses, which is a unit of measure that represents the assumed average maintenance dose per day, of a medicine, when used for its main indication in adults.^16^

There is no consensus regarding which prescribing indicators should be used to measure quality of prescribing in primary care settings.^17^ In the current study the selection of prescribing indicators was based on indicators that have been used in previous studies to estimate the quality of prescribing in primary care.^12^^,^^18^^–^20 High levels of opioid, anxiolytic, antibiotic, and broad-spectrum antibiotic prescribing are commonly used examples of low-value care.^21^ The total number of prescriptions per patient is often used as an indicator to measure the extent of polypharmacy present in practice populations,^10^ and the total cost of medicines can reflect the efforts of pharmacists during medication reviews to either stop medicines, undertake generic substitution, or substitution to cheaper medicines with the same clinical indications.^22^ It is also known that there is variation in these prescribing indicators among the English population according to different population characteristics such as deprivation, age, and gender.^12^^,^^19^^,^^20^^,^^23^

### Statistical analysis

The goal was to estimate changes in average differences in prescribing outcomes that materialise in general practices that hire a clinical pharmacist, compared with the general practices that did not. A two-way fixed-effects (TWFE) regression approach was used to compare differences in prescribing indicators in practices with and without pharmacists following implementation. TWFE regression is a commonly used method for estimating treatment effects with variability in treatment timing using observational (panel) data (in this case the addition of clinical pharmacist roles to the general practice team).^24^ TWFE can account for differences between practices in each time period, and time-invariant confounding between practices using fixed effects.

The study’s treatment was defined as having at least one headcount clinical pharmacist active within a general practice, irrespective of how many hours they work within the practice. A range of controls were included within the regression model to adjust for differences in patient population (age–gender structure, quintile of patient-weighted Index of Multiple Deprivation [income component], and practice population size), workforce composition (GP full-time equivalents [FTEs] per 1000 patients, nurse FTEs per 1000 patients, and FTEs of direct patient care staff excluding pharmacists per 1000 patients), and practice characteristics (dispensing practices and contracting model). FTE measures how many total full-time employees or part-time employees add up to full-time employees for each staff group. In this study the workforce controls were lagged by one-quarter as their levels are likely to inform employment decisions in the following quarter. All analyses were conducted using Stata (version 18).

As a robustness check, in the current study the authors also examined whether the findings changed when classifying practices according to number of clinical pharmacists per practice. This was achieved by splitting the sample of (treated) general practices into tertiles based on the number of FTE pharmacists per 1000 patients for each practice.

## Results

### Descriptive statistics

**Figure 1.**
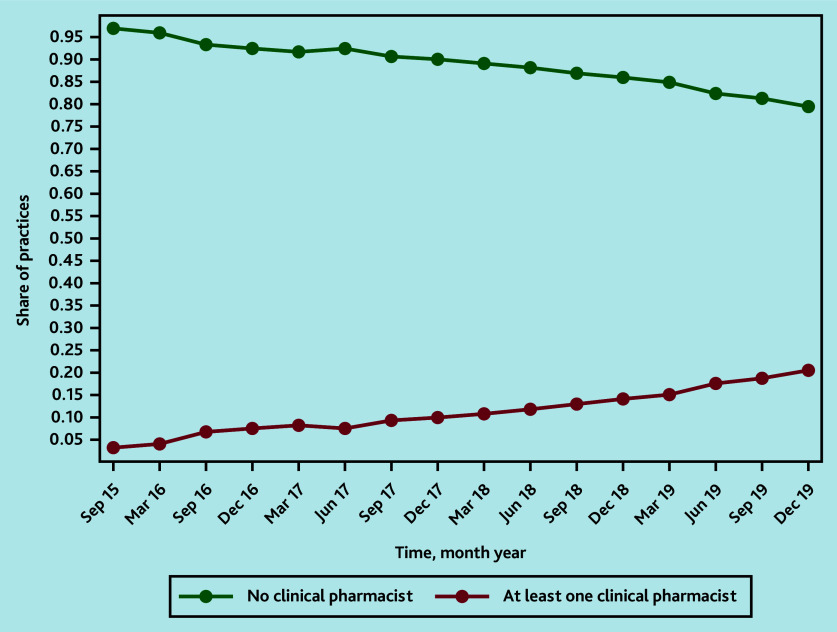
Share of control and treatment practices over time.

Between September 2015 and December 2019, the proportion of practices employing a clinical pharmacist increased from 236/7623 (3.1%) to 1402/6836 (20.5%) (Figure 1). The reduction in the number of GP practices has been noted in previous analyses.^25^

Variation in the number of pharmacists per 1000 patients was also seen (see Supplementary Figure S1). When splitting GP practices into tertiles based on number of pharmacists per 1000 patients, the first tertile has between 0.000 and 0.047 pharmacists per 1000 patients, the second tertile has between 0.047 and 0.077 pharmacists per 1000 patients, and the third tertile has between 0.077 and 0.156 pharmacists per 1000 patients.

There were only small differences in the average share of female patients, level of deprivation, and age breakdown of patient populations in GP practices that adopted a pharmacist and those that did not (Table 1). There were also only small differences in the average contract status of GP practices (general medical services versus alternative provider medical services versus personal medical services contracts) between those with and without clinical pharmacists. Practices that adopted clinical pharmacists were, on average, larger than those that did not during this study’s period of analysis as they had higher numbers of registered patients (10 585 versus 7308 patients). There were also notable differences in the average number of other staff FTE per 1000 patients. Practices that implemented a clinical pharmacist during the study period also had higher average numbers of GP FTEs per 1000 patients (5.73 versus 4.00), nurse FTEs per 1000 patients (2.95 versus 1.84), and other staff involved in direct patient care per 1000 patients (1.77 versus 1.10).

**Table 1.** Baseline descriptives for always controls and treated practices with at least one clinical pharmacist^a^

**Characteristic**	**Control**		**Treated**		**Difference**	

	**Mean**	**SD**	**Mean**	**SD**	**Difference**	***P*-value**
**Practice characteristics**						
Share of patients by age group, %, years						
0–4	5.81	1.53	5.91	1.56	−0.10	0.0203
5–14	11.54	2.37	11.61	2.35	−0.07	0.3292
15–44	38.87	8.31	39.22	8.75	−0.36	0.1487
45–64	26.13	4.03	25.66	4.09	0.47	0.0001
65–74	9.73	3.48	9.67	3.55	0.06	0.5661
75–84	5.62	2.14	5.59	2.15	0.03	0.6493
≥85	2.30	1.08	2.33	1.07	−0.03	0.4105
Share of female patients, %	50.05	2.04	50.30	1.63	−0.25	<0.0001
Total patient population	7308	3896	10 585	5476	−3277	<0.0001
GP FTE in previous month	4.00	2.54	5.73	3.50	−1.73	<0.0001
Nurse FTE in previous month	1.84	1.37	2.95	2.24	−1.11	<0.0001
Other DPC FTE in previous month	1.10	1.45	1.77	2.00	−0.67	<0.0001
IMD, income, % of practices						
Quintile 1	21.27		19.85		1.42	0.1178
Quintile 2	20.57		21.22		−0.65	0.5395
Quintile 3	19.46		20.97		−1.51	0.3006
Quintile 4	19.02		21.16		−2.14	0.0221
Quintile 5	19.69		16.80		2.88	0.0183
GP contract, % of practices						
APMS	2.52		2.24		0.28	0.5987
GMS	66.90		68.33		−1.42	0.4230
PMS	24.81		27.81		−3.00	0.0297
Unknown	5.76		1.62		4.14	<0.0001

**Raw prescribing indicators**						
Total costs of medicines per 1000 patients, £	12 311	3276	12 266	3084	45	0.6371
Total number of prescriptions per 1000 patients, items	1633	537	1616	499	17	0.2851
Total number of antibiotic prescriptions per 1000 patients, items	55	23	54	15	1	0.0964
Share of broad-spectrum versus narrow-spectrum antibiotics, %	8.29	3.71	7.90	3.02	0.39	0.0002
Total number of opioid prescriptions per 1000 patients, items	64	31	64	28	0	0.7178
Oral morphine equivalence of high-dose opioids per 1000 patients, mg	145	103	135	83	10	0.0008
Average daily quantity of anxiolytics per 1000 patients	338	213	317	176	20	0.0008

	***n***		***n***		***N***	

**Observations**	4733		1532		6265	

*Control practices never adopt a clinical pharmacist. Treated practices adopt a clinical pharmacist during our period of analysis.*

a *T-tests were used to ascertain if the difference between treatment and controlled groups was statistically significant. APMS = alternative provider medical services. DPC = direct patient care staff excluding pharmacy-related roles. FTE = full-time equivalent. GMS = general medical services. IMD = Index of Multiple Deprivation. PMS = personal medical services.*

When focusing on unadjusted baseline differences in prescribing indicators between practices that adopted a pharmacist versus those that did not, it can be seen that adopting practices had, on average, reduced oral morphine equivalence of high-dose opioids per 1000 patients (145 mg versus 135 mg), ADQs of anxiolytics per 1000 patients (338 versus 317), and share of broad-spectrum versus narrow-spectrum antibiotics (8.29% versus 7.90%). Although these differences were not statistically significant, adopting practices also had, on average, reduced numbers of total prescriptions per 1000 patients (1633 versus 1616 items), antibiotic prescriptions per 1000 patients (55 versus 54 items), and total costs of medicines per 1000 patients (£12 311 versus £12 266) (Table 1).

### Main analysis

Clinical pharmacist implementation resulted in statistically significant reductions in total costs of medicines per 1000 patients (−0.85%, 95% confidence interval [CI] = −1.50% to −0.21%), the total number of opioid prescriptions per 1000 patients (−1.06%, 95% CI = −1.82% to −0.29%), and the ADQs of anxiolytics per 1000 patients (−1.26%, 95% CI = −2.40% to −0.12%). Clinical pharmacist implementation also resulted in reductions in the total number of prescriptions per 1000 patients (−0.58%, 95% CI = −1.30% to 0.13%), and the total number of antibiotic prescriptions per 1000 patients (−0.51%, 95% CI = −1.30% to 0.27%) that trended towards statistical significance. There were no statistically significant differences in the share of broad-spectrum versus narrow-spectrum antibiotics (0.02%, 95% CI = −0.07% to 0.11%) and the oral morphine equivalence of high-dose opioids (>120 mg per 24 h) per 1000 patients (1.19%, 95% CI = −0.46% to 2.85%). Full regression results, including coefficients for covariates, are included in Supplementary Table S2.

### Supplementary analyses

**Figure 2. fig2:**
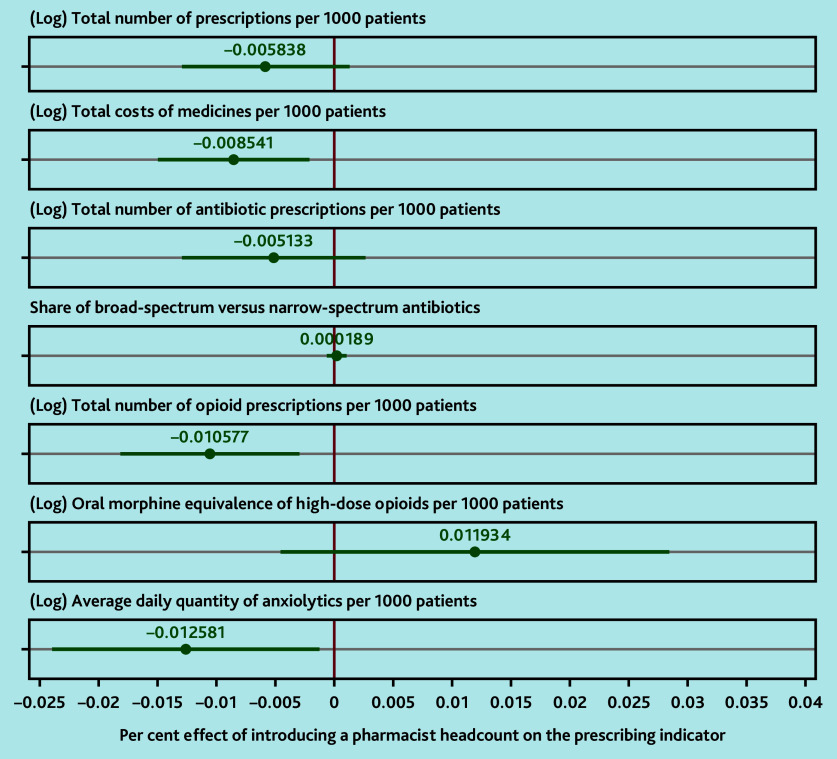
Effect of introducing a pharmacist headcount on practice prescribing. All models were estimated using two-way (time and unit) fixed effects, using practices with a zero headcount of pharmacists as control units. Standard errors were clustered at the level of GP practices. Dots represent point estimates, and lines around them represent the 95% confidence intervals. All models included the following control variables: share of patients in 5-year age–gender bands (0–4-year-old males is the omitted reference group); total number of patients registered with the GP practice; GP FTE in previous month; nurse FTE in previous month; FTE of other direct patient care staff (excluding pharmacy-related roles) in previous month; type of GP practice contract; quintile of IMD (income component) weighted by practice patient population. Full regression results are included in Supplementary Table S2. FTE = full-time equivalent. IMD = Index of Multiple Deprivation.

The results for the supplementary analyses that focused on higher and lower numbers of pharmacists per 1000 patients are reported in Supplementary Tables S3–S5. These demonstrated that the findings are primarily driven by the GP practices within the tertile with the highest number of pharmacists per 1000 patients (see Supplementary Figure S2). In these GP practices, there are larger statistically significant reductions than in the primary analysis for total number of prescriptions per 1000 patients (−2.02%, 95% CI = −3.90% to −0.14%), total costs of medicines per 1000 patients (−2.88%, 95% CI = −4.54% to −1.21%), total number of antibiotic prescriptions per 1000 patients (−2.71%, 95% CI = −4.61% to 0.82%), total number of opioid prescriptions per 1000 patients (−3.40%, 95% CI = −5.41% to −1.38%), and the ADQs of anxiolytics per 1000 patients (−4.25%, 95% CI = −6.72% to −1.77%) (see Supplementary Table S5).[Fig fig2]

The further supplementary analysis that included pharmacy technicians and advanced pharmacy practitioners within the study’s definition of pharmacy staff did not change the overall findings and which prescribing indicators were statistically significant (see Supplementary Table S6).

## Discussion

### Summary

This research demonstrates changes in a broad range of prescribing indicators following the introduction of a clinical pharmacist in general practice. Significant results include reduced prescribing costs and reductions in total number of items, opioids, antibiotics, and anxiolytics prescribed. This supports the hypothesis that clinical pharmacist implementation results in some improvements in quality of prescribing and patient safety in primary care settings. There were no statistically significant differences in the share of broad-spectrum versus narrow-spectrum antibiotics, and the oral morphine equivalence of high-dose opioids between practices that implemented clinical pharmacists versus those that did not.

### Strengths and limitations

A major strength of the current analysis was a methodological approach that attempted to remove confounding by exploiting variation in the timing of implementation of clinical pharmacists across GP practices. Despite this, there are some limitations of this analysis that need to be acknowledged when interpreting the findings.

First, this analysis was focused on aggregate prescribing indicators at the practice level. This can overlook the impact of clinical pharmacists on individual patients, which is important as there is evidence of a positive impact of medication reviews by clinical pharmacists on quality of prescribing for individual patients.^26^^,^^27^

Second, the current analysis did not examine how the roles and responsibilities of individual clinical pharmacists varied within and between practices and the associated impact on quality of prescribing. Unfortunately, this was not possible as no national data collections exist describing this information.

Third, although the study adjusted for different population and workforce factors between treatment and control practices to the extent this was possible, it was not possible to account for unobservable differences in medical complexity of patients registered between treatment and control practices that are not captured by age and gender.

Fourth, the current analysis does not account for other policy developments at the national or local level, such as quality improvement and incentive programmes targeted towards improved prescribing.^28^ It is possible that practices that prioritised implementation of these schemes may also be more likely to employ pharmacists and therefore this could have influenced the results. In light of these points, the authors refrain from attaching a clear causal interpretation to the current results.

Fifth, it was not possible to account for the activity of pharmacists employed by local commissioning bodies (that is, clinical commissioning groups), who can provide prescribing advice and medicines management services to practices. This is because there is no way of attributing their activity within individual practices.

Finally, it can be argued that further insights could be gained by examining a broader range of prescribing indicators. For example, there are a range of further prescribing indicators used by Open Prescribing (https://openprescribing.net), NHS BSA,^29^ and within the NHS England Quality Premium.^28^ However, as mentioned in the current study, there is no consensus on what prescribing indicators should be used to assess quality of prescribing at the GP practice level.^17^ Although the authors of the current study relied on prescribing indicators that have been commonly used in the existing academic literature, it is acknowledged future research is needed to examine more recently developed indicators.

### Comparison with existing literature

Hayhoe *et al* focused on the impact of integrating pharmacists into primary care teams on health system indicators.^9^ The two most examined prescribing indicators were the total number of medications and medication costs per patient. Eleven studies focused on the impact of clinical pharmacist implementation on the total number of medications,^26^^,^^30^^–^39 with four studies showing small statistically significant reductions,^26^^,^^31^^,^^32^^,^^37^ two studies showing increases,^35^^,^^36^ and five studies showing no statistically significant effect.^30^^,^^33^^,^^34^^,^^38^^,^^39^ Twelve studies examined medication costs,^31^^,^^33^^–^35^,^^37^^,^^39^^–^45 with only three studies showing a statistically significant reduction in medication costs associated with pharmacist implementation.^31^^,^^37^^,^^45^ However, only three of these studies are from the UK (two of which showed reductions in medication costs),^35^^,^^43^^,^^44^ with the majority of studies from the US, which may be less applicable to the UK context.

Croke *et al* undertook a systematic review and meta-analysis that examined the impact of integrating clinical pharmacists within general practice on the number of medications prescribed and potentially inappropriate prescribing (PIP) for patients with polypharmacy.^10^ Nine studies focused on the number of medications prescribed per patient,^31^^,^^37^^,^^46^^–^52 with reductions in medications seen in eight studies. Eleven studies focused on PIP,^37^^,^^46^^,^^47^^,^^49^^,^^50^^,^^53^^–^58 with 10 studies demonstrating reductions in PIP in comparison with usual care. Although this review provides useful insights into the impact of clinical pharmacist implementation in primary care on quality of prescribing,^10^ in the current study the authors were unable to analyse these metrics for patients with polypharmacy as aggregate population-level data were analysed across GP practices rather than patient-level data.

### Implications for research and practice

The current research provides evidence that supports the ongoing policy in England of expanding clinical pharmacists working within multidisciplinary teams in general practice. However, further research is needed to establish the exact mechanisms by which clinical pharmacists influence quality of prescribing for different indicators. For example, it is notable that clinical pharmacist implementation was associated with reductions in total antibiotic prescribing as clinical pharmacists typically do not manage acute illnesses that require antibiotics. It is possible that the presence of clinical pharmacists within general practice may influence antibiotic prescribing through other mechanisms such as antimicrobial stewardship interventions, education of GPs, or medication reviews of antibiotics on repeat prescription.^59^

There are also several other potential implications of clinical pharmacist implementation in primary care that should be the focus of future research. At the individual patient level, there has already been significant work establishing the positive impact of pharmacist-led interventions in primary care to reduce medical errors in primary care,^60^^–^62 and community pharmacists to improve medical adherence.^63^^,^^64^ However, more work could be undertaken to establish the specific implications of expanding the number of clinical pharmacists working in multidisciplinary teams in English general practices on medication adherence, patient satisfaction, and polypharmacy.

At the practice level, further research is also needed into the broader implications of pharmacist implementation on demand for primary care services, including utilisation of appointments for different primary care staff types. At the health system level, more could be done to understand how the implementation of clinical pharmacy roles in primary care has an impact on other services such as emergency department attendance or admissions to hospital. Bringing evidence together from the practice and health system-level perspectives would help facilitate cost-effectiveness studies to establish to what extent investment in clinical pharmacists in primary care is warranted versus other staff or interventions.

Finally, further research is needed to establish to what extent changes in clinical pharmacist employment processes, which have moved away from direct employment from general practice to employment within PCNs in recent years, has maintained these improvements in prescribing.

## Data

General practice workforce data were retrieved from NHS England Primary Care Workforce Quarterly Update. Accessible here: https://digital.nhs.uk/data-and-information/publications/statistical/primary-care-workforce-quarterly-update/30-september-2023. Practice characteristics were retrieved from the Office for Health Improvement and Disparities national general practice profiles. Accessible here: https://fingertips.phe.org.uk/profile/general-practice. Prescribing data were retrieved from the NHS Business Services Authority prescribing dataset. Accessible here: https://www.nhsbsa.nhs.uk/prescription-data/prescribing-data/english-prescribing-data-epd.

## References

[1] NHS England (2016). General practice forward view.

[2] NHS England (2019). Investment and evolution: a five-year framework for GP contract reform to implement The NHS Long Term Plan.

[3] NHS England, NHS Improvement (2019). Network contract directed enhanced service: Additional Roles Reimbursement Scheme guidance.

[4] NHS England (2023). Primary care workforce quarterly update, 30 September 2023, experimental statistics. https://digital.nhs.uk/data-and-information/publications/statistical/primary-care-workforce-quarterly-update/30-september-2023.

[5] NHS England (2023). Additional roles: a quick reference summary. https://www.england.nhs.uk/long-read/additional-roles-a-quick-reference-summary.

[6] Bradley F, Seston E, Mannall C, Cutts C (2018). Evolution of the general practice pharmacist’s role in England: a longitudinal study. Br J Gen Pract.

[7] Claire M, Claire A, Matthew B (2022). The role of clinical pharmacists in general practice in England: Impact, perspectives, barriers and facilitators. Res Soc Adm Pharm.

[8] Baird B, Beech J (2020). Community pharmacy explained. https://www.kingsfund.org.uk/insight-and-analysis/long-reads/community-pharmacy-explained.

[9] Hayhoe B, Cespedes JA, Foley K (2019). Impact of integrating pharmacists into primary care teams on health systems indicators: a systematic review. Br J Gen Pract.

[10] Croke A, Cardwell K, Clyne B (2023). The effectiveness and cost of integrating pharmacists within general practice to optimize prescribing and health outcomes in primary care patients with polypharmacy: a systematic review. BMC Prim Care.

[11] Mann C, Anderson C, Avery AJ (2018). Clinical pharmacists in general practice: pilot scheme Independent evaluation report: full report.

[12] Francetic I, Gibson J, Spooner S (2022). Skill-mix change and outcomes in primary care: Longitudinal analysis of general practices in England 2015–2019. Soc Sci Med.

[13] Office for Health Improvements and Disparities (2024). National general practice profiles. https://fingertips.phe.org.uk/profile/general-practice.

[14] Street KA, Taylor ADJ (2023). A consensus building study to define the role of a ‘clinical’ pharmacy technician in a primary care network environment in England. Int J Pharm Pract.

[15] Martin S, Shaw N, Burnage K, Petty D (2022). Role of advanced practice pharmacists in general practice. Prescriber.

[16] Walley T, Roberts D (2000). Average daily quantities: a tool for measuring prescribing volume in England. Pharmacoepidemiol Drug Saf.

[17] Barber N (1995). What constitutes good prescribing?. BMJ.

[18] Radomski TR, Decker A, Khodyakov D (2022). Development of a metric to detect and decrease low-value prescribing in older adults. JAMA Netw Open.

[19] Hodkinson A, Zghebi SS, Kontopantelis E (2023). Association of strong opioids and antibiotics prescribing with GP burnout: a retrospective cross-sectional study. Br J Gen Pract.

[20] Allen T, Gyrd-Hansen D, Kristensen SR (2022). Physicians under pressure: evidence from antibiotics prescribing in England. Med Decis Making.

[21] Colla CH, Mainor AJ, Hargreaves C (2017). Interventions aimed at reducing use of low-value health services: a systematic review. Med Care Res Rev.

[22] Dalton K, Byrne S (2017). Role of the pharmacist in reducing healthcare costs: current insights. Integr Pharm Res Pract.

[23] Curtis HJ, Croker R, Walker AJ (2019). Opioid prescribing trends and geographical variation in England, 1998–2018: a retrospective database study. Lancet Psychiatry.

[24] Wooldridge JM (2021). Two-way fixed effects, the two-way Mundlak regression, and difference-in-differences estimators. SSRN.

[25] Hutchinson J, Checkland K, Gibson J (2023). Consequences of the closure of general practices: a retrospective cross-sectional study. Br J Gen Pract.

[26] Lenander C, Elfsson B, Danielsson B (2014). Effects of a pharmacist-led structured medication review in primary care on drug-related problems and hospital admission rates: a randomized controlled trial. Scand J Prim Health Care.

[27] Lexow M, Wernecke K, Sultzer R (2022). Determine the impact of a structured pharmacist-led medication review -a controlled intervention study to optimise medication safety for residents in long-term care facilities. BMC Geriatr.

[28] Bou-Antoun S, Costelloe C, Honeyford K (2018). Age-related decline in antibiotic prescribing for uncomplicated respiratory tract infections in primary care in England following the introduction of a national financial incentive (the Quality Premium) for health commissioners to reduce use of antibiotics in the community: an interrupted time series analysis. J Antimicrob Chemother.

[29] NHS Business Services Authority, NHS Digital (2019). Medication safety -indicators specification.

[30] Hunt JS, Siemienczuk J, Pape G (2008). A randomized controlled trial of team-based care: impact of physician-pharmacist collaboration on uncontrolled hypertension. J Gen Intern Med.

[31] Britton ML, Lurvey PL (1991). Impact of medication profile review on prescribing in a general medicine clinic. Am J Hosp Pharm.

[32] Harris IM, Westberg SM, Frakes MJ, Van Vooren JS (2009). Outcomes of medication therapy review in a family medicine clinic. J Am Pharm Assoc.

[33] Okamoto MP, Nakahiro RK (2001). Pharmacoeconomic evaluation of a pharmacist-managed hypertension clinic. Pharmacother.

[34] Sellors J, Kaczorowski J, Sellors C (2003). A randomized controlled trial of a pharmacist consultation program for family physicians and their elderly patients. CMAJ.

[35] Zermansky AG, Petty DR, Raynor DK (2001). Randomised controlled trial of clinical medication review by a pharmacist of elderly patients receiving repeat prescriptions in general practice. BMJ.

[36] Mourão AOM, Ferreira WR, Martins MAP (2013). Pharmaceutical care program for type 2 diabetes patients in Brazil: a randomised controlled trial. Int J Clin Pharm.

[37] Campins L, Serra-Prat M, Gózalo I (2017). Randomized controlled trial of an intervention to improve drug appropriateness in community-dwelling polymedicated elderly people. Fam Pract.

[38] Obreli-Neto PR, Marusic S, Guidoni CM (2015). Economic evaluation of a pharmaceutical care program for elderly diabetic and hypertensive patients in primary health care: a 36-month randomized controlled clinical trial. J Manag Care Spec Pharm.

[39] Sellors C, Dalby DM, Howard M (2001). A pharmacist consultation service in community-based family practices: a randomized, controlled trial in seniors. J Pharm Technol.

[40] Stergachis A, Fors M, Wagner EH (1987). Effect of clinical pharmacists on drug prescribing in a primary-care clinic. Am J Hosp Pharm.

[41] Finley PR, Rens HR, Pont JT (2003). Impact of a collaborative care model on depression in a primary care setting: a randomized controlled trial. Pharmacother.

[42] Borenstein JE, Graber G, Saltiel E (2003). Physician-pharmacist comanagement of hypertension: a randomized, comparative trial. Pharmacother.

[43] Neilson AR, Bruhn H, Bond CM (2015). Pharmacist-led management of chronic pain in primary care: costs and benefits in a pilot randomised controlled trial. BMJ Open.

[44] Phelan M, Foster NE, Thomas E (2008). Pharmacist-led medication review for knee pain in older adults: content, process and outcomes. Int J Pharm Pract.

[45] Siaw MYL, Ko Y, Malone DC (2017). Impact of pharmacist-involved collaborative care on the clinical, humanistic and cost outcomes of high-risk patients with type 2 diabetes (IMPACT): a randomized controlled trial. J Clin Pharm Ther.

[46] Hanlon JT, Weinberger M, Samsa GP (1996). A randomized, controlled trial of a clinical pharmacist intervention to improve inappropriate prescribing in elderly outpatients with polypharmacy. Am J Med.

[47] Taylor CT, Byrd DC, Krueger K (2003). Improving primary care in rural Alabama with a pharmacy initiative. Am J Health-Syst Pharm.

[48] Lenaghan E, Holland R, Brooks A (2007). Home-based medication review in a high risk elderly population in primary care – the POLYMED randomised controlled trial. Age Ageing.

[49] Verdoorn S, Kwint H-F, Blom JW (2019). Effects of a clinical medication review focused on personal goals, quality of life, and health problems in older persons with polypharmacy: a randomised controlled trial (DREAMeR-study). PLoS Med.

[50] Vinks THAM, Egberts TCG, de Lange TM, de Koning FHP (2009). Pharmacist-based medication review reduces potential drug-related problems in the elderly: the SMOG controlled trial. Drugs Aging.

[51] Bernsten C, Björkman I, Caramona M (2001). Improving the well-being of elderly patients via community pharmacy-based provision of pharmaceutical care: a multicentre study in seven European countries. Drugs Aging.

[52] Jódar-Sánchez F, Malet-Larrea A, Martín JJ (2015). Cost-utility analysis of a medication review with follow-up service for older adults with polypharmacy in community pharmacies in Spain: the conSIGUE program. Pharmacoeconomics.

[53] Granås AG, Bates I (1999). The effect of pharmaceutical review of repeat prescriptions in general practice. Int J Pharm Pract.

[54] Krska J, Cromarty JA, Arris F (2001). Pharmacist-led medication review in patients over 65: a randomized, controlled trial in primary care. Age Ageing.

[55] Geurts MME, Stewart RE, Brouwers JRBJ (2016). Implications of a clinical medication review and a pharmaceutical care plan of polypharmacy patients with a cardiovascular disorder. Int J Clin Pharm.

[56] van der Meer HG, Wouters H, Pont LG, Taxis K (2018). Reducing the anticholinergic and sedative load in older patients on polypharmacy by pharmacist-led medication review: a randomised controlled trial. BMJ Open.

[57] Sloeserwij VM, Hazen ACM, Zwart DLM (2019). Effects of non-dispensing pharmacists integrated in general practice on medication-related hospitalisations. Br J Clin Pharmacol.

[58] Bryant LJM, Coster G, Gamble GD, McCormick RN (2011). The General Practitioner-Pharmacist Collaboration (GPPC) study: a randomised controlled trial of clinical medication reviews in community pharmacy. Int J Pharm Pract.

[59] Saha SK, Hawes L, Mazza D (2019). Effectiveness of interventions involving pharmacists on antibiotic prescribing by general practitioners: a systematic review and meta-analysis. J Antimicrob Chemother.

[60] Avery AJ, Rodgers S, Cantrill JA (2012). A pharmacist-led information technology intervention for medication errors (PINCER): a multicentre, cluster randomised, controlled trial and cost-effectiveness analysis. Lancet.

[61] Elliott RA, Putman KD, Franklin M (2014). Cost effectiveness of a pharmacist-led information technology intervention for reducing rates of clinically important errors in medicines management in general practices (PINCER). Pharmacoeconomics.

[62] Rodgers S, Taylor AC, Roberts SA (2022). Scaling-up a pharmacist-led information technology intervention (PINCER) to reduce hazardous prescribing in general practices: multiple interrupted time series study. PLOS Med.

[63] Elliott RA, Boyd MJ, Salema N-E (2016). Supporting adherence for people starting a new medication for a long-term condition through community pharmacies: a pragmatic randomised controlled trial of the New Medicine Service. BMJ Qual Saf.

[64] Elliott RA, Boyd MJ, Tanajewski L (2020). ‘New medicine service’: supporting adherence in people starting a new medication for a long-term condition: 26-week follow-up of a pragmatic randomised controlled trial. BMJ Qual Saf.

